# The role of potassium channels in the endothelial dysfunction induced by periodontitis

**DOI:** 10.1590/1678-7757-2018-0048

**Published:** 2018-10-04

**Authors:** Luiz Renato Olchanheski, Regina Sordi, Junior Garcia Oliveira, Gustavo Ferreira Alves, Reila Taina Mendes, Fábio André Santos, Daniel Fernandes

**Affiliations:** 1 Universidade Estadual de Ponta Grossa, Departamento de Ciências Farmacêuticas, Ponta Grossa, Paraná, Brasil.; 2 Universidade Estadual de Ponta Grossa, Departamento de Biologia Molecular e Genética, Ponta Grossa, Paraná, Brasil.; 3 Universidade Federal de Santa Catarina, Departamento de Farmacologia, Florianópolis, Santa Catarina, Brasil.; 4 Universidade Estadual de Ponta Grossa, Departamento de Odontologia, Ponta Grossa, Paraná, Brasil.

**Keywords:** Endothelium, Nitric oxide, Periodontitis, Potassium channels

## Abstract

**Objective:**

Periodontitis is associated with endothelial dysfunction, which is clinically characterized by a reduction in endothelium-dependent relaxation. However, we have previously shown that impairment in endothelium-dependent relaxation is transient. Therefore, we evaluated which mediators are involved in endothelium-dependent relaxation recovery.

**Material and methods:**

Rats were subjected to ligature-induced experimental periodontitis. Twenty-one days after the procedure, the animals were prepared for blood pressure recording, and the responses to acetylcholine or sodium nitroprusside were obtained before and 30 minutes after injection of a nitric oxide synthase inhibitor (L-NAME), cyclooxygenase inhibitor (Indomethacin, SC-550 and NS- 398), or calcium-dependent potassium channel blockers (apamin plus TRAM- 34). The maxilla and mandible were removed for bone loss analysis. Blood and gingivae were obtained for C-reactive protein (CRP) and myeloperoxidase (MPO) measurement, respectively.

**Results:**

Experimental periodontitis induces bone loss and an increase in the gingival MPO and plasmatic CRP. Periodontitis also reduced endothelium-dependent vasodilation, a hallmark of endothelial dysfunction, 14 days after the procedure. However, the response was restored at day 21. We found that endothelium-dependent vasodilation at day 21 in ligature animals was mediated, at least in part, by the activation of endothelial calcium-activated potassium channels.

**Conclusions:**

Periodontitis induces impairment in endothelial-dependent relaxation; this impairment recovers, even in the presence of periodontitis. The recovery is mediated by the activation of endothelial calcium-activated potassium channels in ligature animals. Although important for maintenance of vascular homeostasis, this effect could mask the lack of NO, which has other beneficial properties.

## Introduction

Periodontitis is a chronic inflammatory disease that compromises the integrity of tooth-supporting tissues. In addition to the local effects of this disease, periodontitis is also associated with systemic inflammation. ^2^ ^,^ ^17^ Furthermore, periodontitis has been associated with endothelial dysfunction, ^15^ ^,^ ^23^ ^,^ ^33^ which is an early event in the development of cardiovascular diseases, especially atherosclerosis. ^34^

One of the hallmarks of endothelial dysfunction is a reduced response to endothelial-dependent stimuli, such as acetylcholine. Acetylcholine stimulates endothelial nitric oxide synthase (NOS-3) to generate nitric oxide (NO), which diffuses to the underlying smooth muscle cells, inducing relaxation by increasing the production of cGMP, leading to a transient reduction in blood pressure *in vivo* . ^2^ Thus, endothelial dysfunction is broadly defined as an impairment in vascular relaxation, due to decreased NO production by the endothelium and/or increased inactivation of NO. ^14^ Besides being a potent vasodilator, NO has antithrombotic, anti-inflammatory, and antimitogenic properties, which explains why the reduction in NO levels is associated with increased cardiovascular disease risk. ^21^

**Figure 1:**
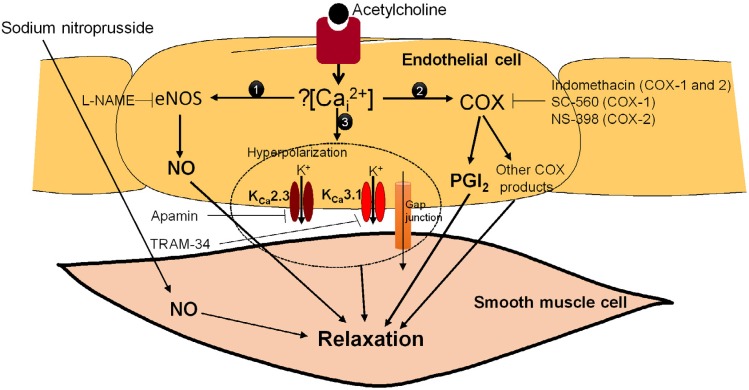
Endothelium-dependent vasodilation. Acetylcholine induces an increase in intracellular calcium concentration and leads to the production and release of nitric oxide (NO) and prostacyclin (PGI_2_), in addition to the opening of potassium channels, which in turn induces vascular smooth muscle cell relaxation. NO diffuses to the underlying smooth muscle cell and induces relaxation by increasing the production of cGMP. Prostacyclin is the main prostanoid synthesized by vascular endothelium, and acts via the prostacyclin receptor (IP). The opening of intermediate conductance calcium-activated potassium channels (Kca3.1) and small conductance calcium-activated potassium channels (Kca2.3) in the endothelial cell induces vascular smooth muscle cell hyperpolarization, which is evoked by electrical coupling through the myoendothelial gap junctions

However, the perception of endothelial dysfunction as just a reduction in NO production/bioavailability is oversimplified. *In vitro* experiments have shown that, in large conduit vessels such as the aorta, acetylcholine-induced vasodilatation is predominantly mediated by NO. ^29^ However, in addition to NO, other mediators such as prostacyclin (PGI_2_) and endothelium-dependent hyperpolarization (EDH) contribute to endothelium-dependent vasodilator response to agonists in small resistance vessels. ^1^ Prostacyclin is a product of the metabolism of arachidonic acid by cyclooxygenase (COX), and acts through the prostacyclin receptor (IP). ^18^ EDH has been proposed to mediate vasodilation through the initial activation of small conductance (K_Ca_2.3) and intermediate conductance (K_Ca_3.1) calcium-activated potassium channels, which are present on the endothelium. ^10^ Following the opening of the K_Ca_2.3 and K_Ca_3.1 channels in the endothelial cell, vascular smooth muscle cell hyperpolarization is evoked by electrical coupling through the myoendothelial gap junction ( Figure 1 ). ^7^

Interestingly, a compensatory increase in EDH and/or prostacyclin-mediated vasodilation in response to acetylcholine has been demonstrated in blood vessels of NOS-3 knockout mice. ^12^ ^,^ ^19^ ^,^ ^32^ Therefore, there is a redundancy in the system, and more than one endothelial mediator is capable of acting as the signal between endothelium and smooth muscle. This could explain why some studies have reported normal endothelium-dependent relaxation in models of atherosclerosis. ^25^

We have previously demonstrated a reduction in acetylcholine response 14 days after ligature-induced periodontitis. ^2^ However, despite lasting systemic and vascular inflammation, impairment in the acetylcholine response is restored 21 days onwards after the procedure. ^2^ ^,^ ^24^ Since endothelium-dependent relaxation is the main mechanism for assessing endothelial dysfunction, ^5^ the compensatory effect could mask a lack of NO and an increased risk of cardiovascular disease.

Therefore, the main aim of this study was to assess the involvement of nitric oxide, potassium channels, and COX products in the restoration of the endothelium-dependent response during periodontitis.

## Material and methods

### Compounds

Acetylcholine, sodium nitroprusside, apamin, TRAM-34, SC-560, NS-398, indomethacin, methylene blue, hexadecyltrimethylammonium bromide (HTAB), 3,3',5,5'-tetramethylbenzidine (TMB), dimethylsulfoxide (DMSO) and N_ω_-Nitro-L-arginine methyl ester hydrochloride (L-NAME) were purchased from Sigma Chemical Co. (Sigma-Aldrich; St Louis, MO, USA). Ketamine was obtained from Parke-Davis (Parke-Davis; São Paulo, SP, Brazil) and xylazine from Bayer (Bayer; São Paulo, SP, Brazil). Bradford was purchased from Bio-Rad Laboratories, Inc (Bio-Rad Laboratories Inc.; Hercules, CA, USA). Heparin was a kind gift from Cristália Laboratories (Cristália; São Paulo, SP, Brazil). Acetylcholine, sodium nitroprusside, apamin, and L-NAME were dissolved in sterile phosphate-buffered saline. Indomethacin was dissolved in phosphate-buffered saline with sodium carbonate (5%). TRAM-34, SC-560, and NS-398 were prepared as a concentrated stock solution in DMSO, and were injected at a volume never higher than 0.5 mL/kg. At this dose, DMSO does not significantly change blood pressure. ^31^

### Animals

Male Wistar rats (200-250 g; 10 weeks old) were housed in a temperature- and light-controlled room with free access to water and food. All of the procedures were performed in accordance with the European Communities Council Directive of 24 November 1986 (86/609/EEC) and the Guide of the Brazilian National Council of Animal Experimentation. The procedures were approved by the University's Institutional Ethics Committee (protocol number 019/2013).

### Ligature-induced periodontitis

To induce periodontitis, rats were first anesthetized with an intraperitoneal injection of ketamine and xylazine (90 and 15 mg/kg, respectively). A cotton ligature 4/0 (Roboz Surgical Instrument Co.; Gaithersburg MD, USA) was placed around the cervixes of both sides (right and left) of the mandibular first molars and maxillary second molars in each animal, exactly as previously described. ^2^ Placement of ligatures induces periodontal disease by facilitating gingival bacterial invasion. ^28^ Sham-operated rats had the ligature removed immediately after the procedure.

### Mean arterial pressure measurement

The animals were anesthetized intramuscularly with ketamine and xylazine (90 and 15 mg/kg, respectively, supplemented at 60 min intervals), and heparinized PE-20 and PE-50 polyethylene catheters were inserted into the left femoral vein for drug injections and into the right carotid artery for recording mean arterial pressure, respectively. To prevent clotting, a bolus dose of heparin (300 IU) was injected immediately after vein cannulation. The animals were allowed to breathe spontaneously via a tracheal cannula. The body temperature was monitored by a rectal thermometer and maintained at 36±1°C by means of a heating table. Blood pressure and heart rate data were recorded with a catheter pressure transducer coupled to a Powerlab 4/30 (ADInstruments Pty Ltd.; Castle Hill, New South Wales, Australia) running the proprietary software LabChart 8®.

After stabilization, basal mean arterial pressure was obtained and the animals received intravenous injections containing either acetylcholine (3, 10, and 30 nmol/kg) or sodium nitroprusside (3, 10, and 30 nmol/kg). The doses were injected in a total volume of 250 μL (including washing of the catheter). An interval of 8 min was allowed between each administration for mean arterial pressure stabilization. The change in mean arterial pressure (in mmHg) was calculated and compared among the groups. At the end of the experiment, the animals were sacrificed with a pentobarbital overdose.

### Myeloperoxidase (MPO)

After the blood pressure experiments, the mandible and maxilla were removed and the gingival tissue around the ligated molars was harvested. MPO, indicating neutrophil infiltration into the tissue, was measured in equal-sized samples for each molar. Briefly, samples were homogenized (1:20 w:v) in ice-cold 20 mM sodium phosphate buffer (pH 7.40). After centrifuging, supernatants were removed and pellets were suspended in HTAB buffer (0.5% in 80 mM sodium phosphate buffer, pH 5.4). Supernatant was assayed for protein content determination (Bradford protein assay) and MPO activity was assessed by measuring the H_2_O_2_-dependent oxidation of TMB. In its oxidized form, TMB has a blue color, which was measured spectrophotometrically at 650 nm. The reaction mixture for analysis consisted of 30 μL of tissue sample, 180 μL of H_2_O_2_ (final concentration 0.3 mM) diluted in 0.08 M phosphate buffer, pH 5.4, and 30 μL of TMB (final concentration 0.16 mM) dissolved in DMSO. The reaction was performed in a 96-well microtiter plate. The mixture was incubated for 10 min at 37°C, and optical density (650 nm) was measured every 2 min using a ultra-microplate reader (EL 808, Bio-Tek Instruments Inc.; Winooski, VT, USA). A 5 min incubation time was used, and myeloperoxidase activity was expressed as optical density at 650 nm *per* mg of protein.

### Measurement of C-reactive protein (CRP)

Blood was collected through the catheters inserted in the right carotid artery for recording of mean arterial pressure. Plasma CRP was quantified using a highly sensitive, rat enzyme-linked immunosorbent assay (ELISA) kit (Immunology Consultants Laboratory Inc.; Newberg, OR, USA).

### Measurement of alveolar bone loss

After collecting gingival tissues for MPO assay, the mandible and maxilla were dissected. The defleshed mandibles and maxillae were stained with aqueous 1% methylene blue for identifying the cement-enamel junction. Standardized pictures of each specimen were taken from the lingual and the buccal aspects of the specimens. All images were measured with computer software (ImageJ, available in the public domain by the US National Institutes of Health, Bethesda, MD, USA) by the same blinded and calibrated examiner. The distance of alveolar bone loss was measured between the cement-enamel junction and the alveolar bone crest. Five points were measured along the buccal and lingual portions, and the average was calculated for each molar, as previously described. ^2^

### Experimental protocol

Twenty-four animals were subjected to the ligature procedure. One hour (day 0), 14 days, or 21 days after the procedure, eight animals were randomly prepared for blood pressure measurement. Consecutive dose-response curves to acetylcholine and sodium nitroprusside were obtained. At the end of the experiment, blood samples were collected for CRP measurement. Next, animals were killed, gingival tissues around ligated molars were harvested for MPO assay, and the mandible and maxilla were dissected for alveolar bone loss measurement. In this set of experiments, we opted to perform the control group as ligature day 0 (one hour after ligature procedure) to avoid performing a sham-matched group for each time.

In another set of experiments, 48 animals were divided into two groups of 24 animals each, and subjected to either ligature or sham procedure. Twenty-one days after the procedure, the animals were prepared for blood pressure measurement. After performing dose-response curves to acetylcholine and sodium nitroprusside, the animals received an intravenous injection of L-NAME (nonselective NOS inhibitor; 20 mg/kg), indomethacin (nonselective COX inhibitor; 10 mg/kg), or apamin (K_Ca_2.3 channel inhibitor; 75 μg/kg) plus TRAM-34 (K_Ca_3.1 channel inhibitor; 1 mg/kg), randomly. The control animals received vehicle (see Compounds section). Thirty minutes after drug injection, new dose response curves to acetylcholine and sodium nitroprusside were obtained.

Eighteen animals were subjected to a protocol similar to the one previously described; however, all of them were subjected to the ligature procedure. Twenty-one days after the procedure, the animals were prepared for blood pressure measurement. After performing dose-response curves to acetylcholine, the animals randomly received intravenous injection of SC-560 (selective inhibitor of COX-1; 2 mg/kg), NS-398 (selective inhibitor of COX-2; 2 mg/kg), or SC-560 (2 mg/kg) plus NS-398 (2 mg/kg). The doses of the drugs mentioned above were chosen based on previous studies. ^4^ ^,^ ^8^ ^,^ ^9^ ^,^ ^27^ ^,^ ^30^ ^,^ ^31^

The time of the analysis was based on our previous studies where we demonstrated a reduction in acetylcholine response 14 days after ligature-induced periodontitis and a recovery of response from 21 days, despite the presence of systemic and vascular inflammation. ^2^ ^,^ ^24^

### Statistical analysis

The data were expressed as the mean±SEM of 6-8 rats, as indicated in the figure legends. Statistical significance was analyzed by one or two-way ANOVA, followed by Bonferroni's *post hoc* or Student's *t* tests, as indicated in the figure legends. A *p* -value lesser than 0.05 was considered as statistically significant. All variables presented normal distributions and homogeneity of variances, as indicated by the Shapiro-Wilk and Bartlett tests, respectively.

## Results

### Effect of ligature-induced periodontitis on alveolar bone loss and endothelial function

**Figure 2:**
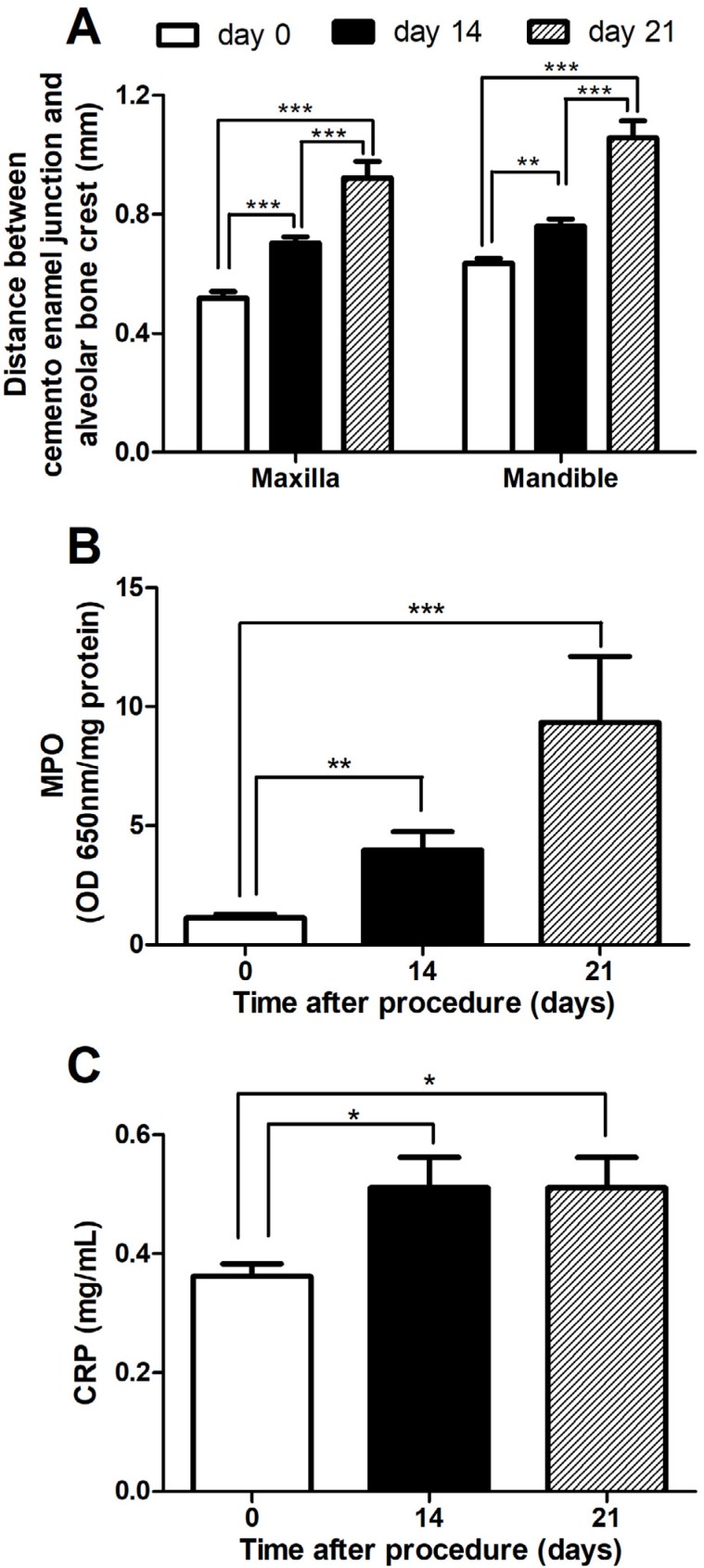
Local and systemic effects induced by periodontitis. Bone loss of the maxilla and mandible was measured on day 0, 14 days, or 21 days after the ligature procedure (A). Bone loss was quantified as the distance between the cement enamel junction and the alveolar crest (in mm). Myeloperoxidase (MPO) activity was also measured in the gingival tissue around the ligated molars (B). Blood was collected and assayed for C-reactive protein (CRP) (C). Statistical analysis was performed using one-way analysis of variance followed by Bonferroni *post hoc* test. *p<0.05, **p<0.01, and ***p<0.001

The ligature was placed around the second maxillary molars and the first mandibular molars on both sides (right and left). However, for the sake of clarity, we pooled the results from the right and left maxilla and mandibles ( Figure 2A ). Alveolar bone loss was observed in the maxillary and mandible molars in the ligated rats 14 and 21 days after the ligature procedure ( Figure 2A ), showing a progressive bone loss. There was also an increase in MPO levels 14 and 21 days after the ligature procedure ( Figure 2B ). C-reactive protein levels were increased 14 and 21 days after ligature, when compared to day 0 ( Figure 2C ).

**Figure 3:**
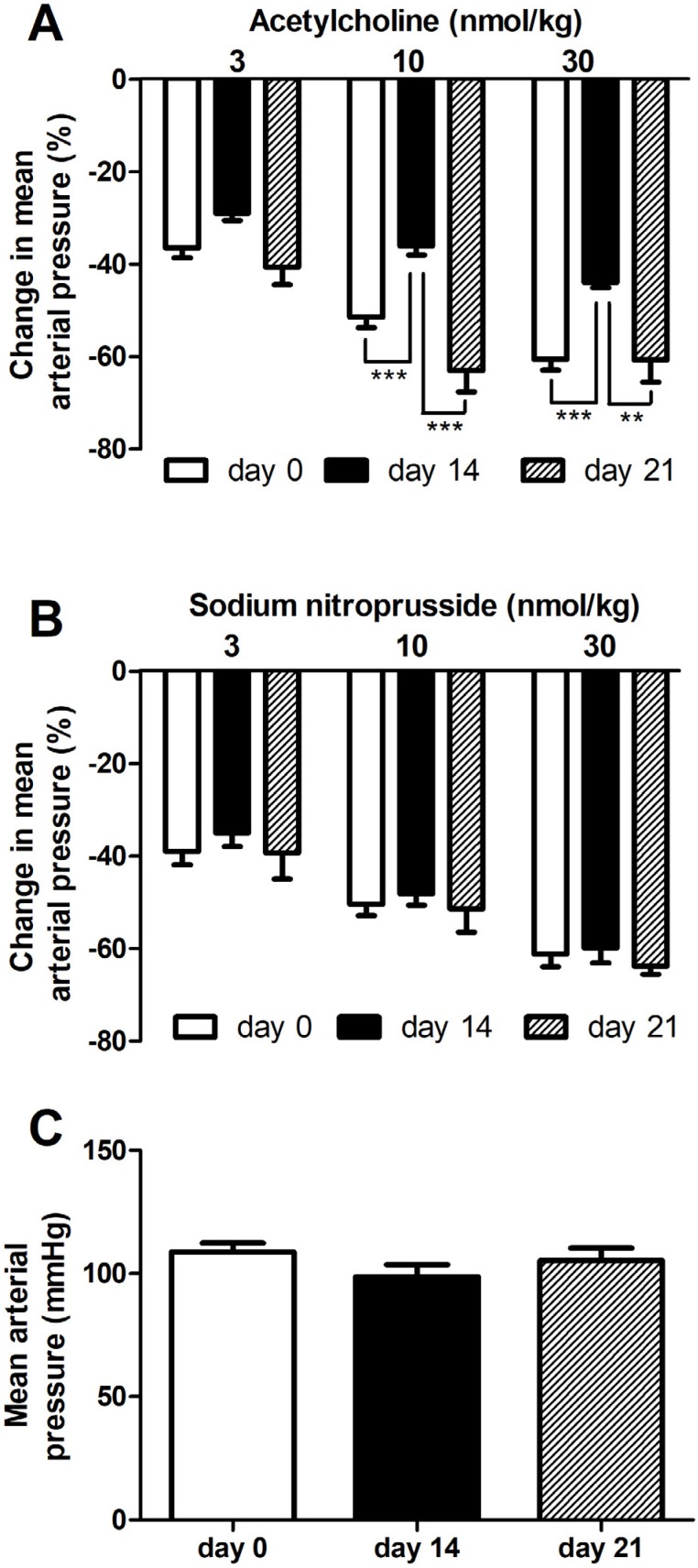
Transient endothelial dysfunction induced by periodontitis. Ligature rats were prepared for mean arterial pressure recording 1 h (day 0), 14 days, or 21 days after the procedure. Increasing doses of acetylcholine (A) or sodium nitroprusside (B) were injected, and the change in mean arterial pressure (% of decrease from basal blood pressure) was determined. The basal mean arterial pressure is also shown (C). Statistical analysis was performed using one-way analysis of variance followed by Bonferroni post *hoc test.* **p<0.01 and *** p<0.001

To evaluate endothelial function in rats with experimental periodontitis, we evaluated the effects of endothelium-dependent and endothelium-independent vasodilators (acetylcholine and sodium nitroprusside, respectively). The effects of the higher doses of acetylcholine were reduced approximately 30% in rats subjected to ligature procedure 14 days earlier ( Figure 3A ). However, the response to acetylcholine was similar to day 0 in rats submitted to ligature 21 days earlier ( Figure 3A ). In contrast, the response to sodium nitroprusside was similar among the groups at all evaluated times ( Figure 3B ). There is no change in basal blood pressure in the times evaluated ( Figure 3C ).

### Involvement of NOS, COX, or EDH restoration of the acetylcholine-induced reduction in blood pressure

Because the main objective of this study was to evaluate the mechanism involved in the restoration of acetylcholine-induced reduction in blood pressure, the next experiments were performed only 21 days after the ligature.

**Figure 4:**
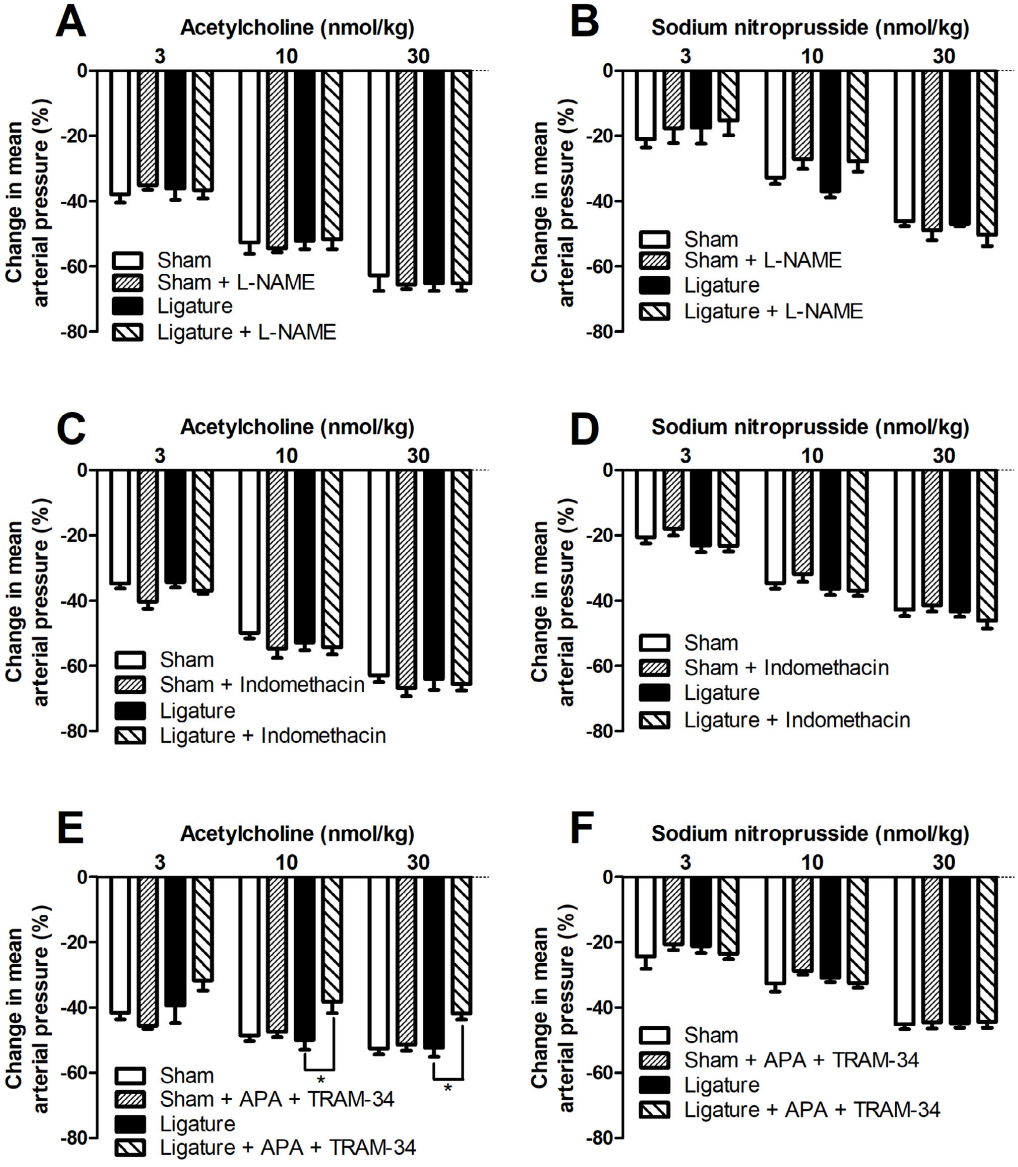
Involvement of NOS, COX, or EDH restoration of the acetylcholine-induced reduction in blood pressure. Twenty-one days after ligature or sham procedure, the animals were prepared for measurement of mean arterial pressure, and dose response curves to acetylcholine and sodium nitroprusside were performed before and 30 min after L-NAME (20 mg/kg, i.v; panels A-B), indomethacin (10 mg/kg, i.v; panels C-D) or apamin plus TRAM-34 (75 μg/kg and 1 mg/kg, respectively, i.v; panels E-F). The change in mean arterial pressure (% of decrease from basal blood pressure) was determined. Statistical analyses were performed using two-way analysis of variance followed by Bonferroni *post hoc* test. *p<0.05

**Figure 5:**
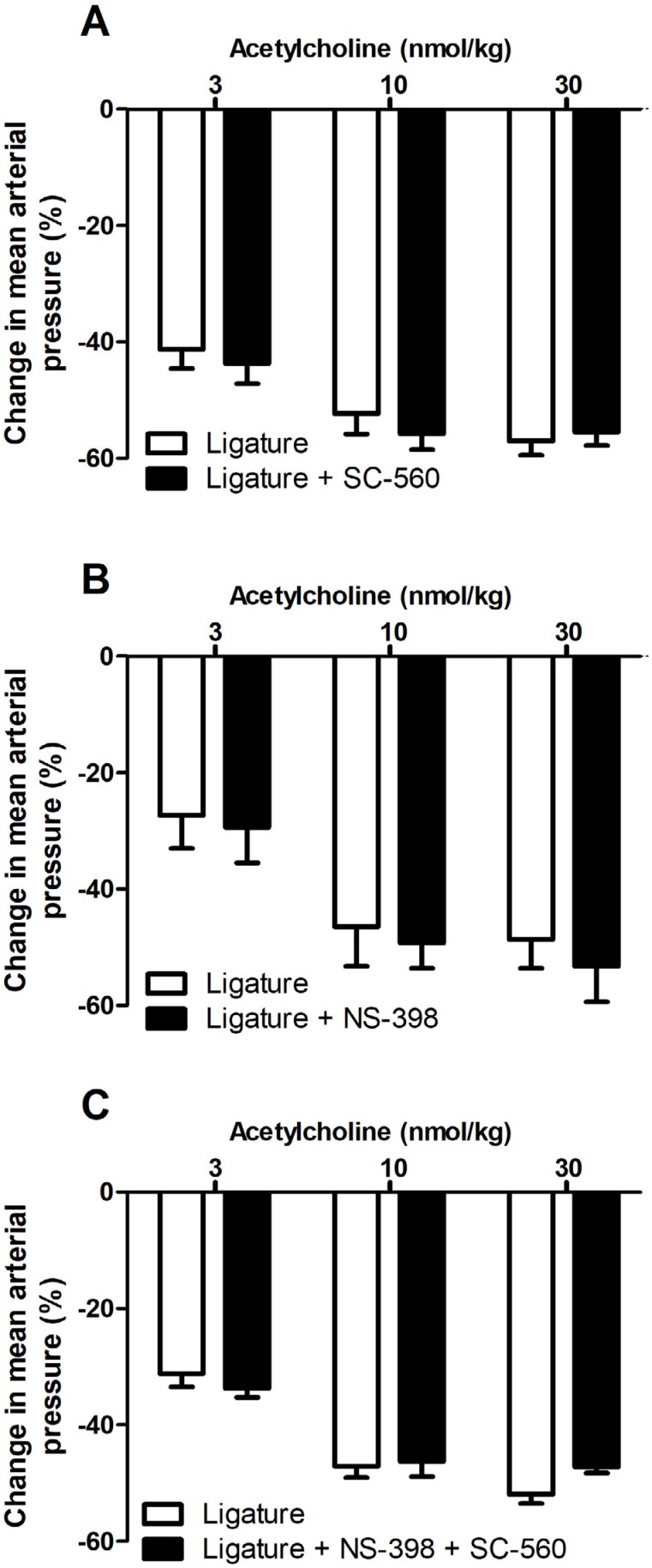
Effect of selective COX inhibitors in the compensatory response to acetylcholine-induced hypotension during periodontitis. Twenty-one days after ligature procedure, the animals were prepared for blood pressure recording and a dose response curve to acetylcholine was performed. Then, SC-560 (selective COX-1 inhibitor; 2 mg/kg; i.v; A), NS-398 (selective COX-2 inhibitor; 2 mg/kg; i.v; B) or NS-398 plus SC-560 (both 2 mg/kg; i.v.; C) were injected. After 30 minutes, a second dose response curve to acetylcholine was obtained. The change of mean arterial pressure (% of decrease from basal blood pressure) was calculated. Statistical analyses were performed using Student's t test for paired samples

The administration of the NOS inhibitor L-NAME in sham or periodontitis rats did not change the peak reduction in mean arterial pressure to sodium nitroprusside or acetylcholine ( Figure 4A-B ). Similarly, the non-selective COX inhibitor, indomethacin, did not change the sodium nitroprusside or acetylcholine response of sham or periodontitis rats ( Figure 4C-D ). In addition, the selective COX-1 inhibitor (SC-560), selective COX-2 inhibitor (NS-398), and both together, did not change acetylcholine response ( Figure 5 ). On the other hand, the blockade of calcium-activated potassium channels (apamin plus TRAM-34) reduced acetylcholine response, only in ligature animals, with no change in the sham response ( Figure 4E ). Apamin plus TRAM-34 did not change sodium nitroprusside response ( Figure 4F ).

L-NAME increased mean arterial pressure, however, there is no statistical difference between sham and ligature (Sham control, 106±4; Sham + L-NAME, 151±5*; Ligature control, 107±9; Ligature + L-NAME*, 142±2; in mmHg, * *p* <0.05 compared to control). Neither indomethacin nor the blockade of calcium-activated potassium channels changed basal blood pressure (data not shown).

## Discussion

In this study, we observed the development of a compensatory endothelium-dependent hypotension in animals with experimental periodontitis, which was, at least in part, mediated by the opening of calcium-activated potassium channels. These findings suggest that endothelial dysfunction is a temporally complex event.

Disturbances in endothelial function leading to reduced endothelium-dependent vasodilation are present in patients with cardiovascular risk factors, including hypertension, hypercholesterolemia, diabetes, smoking, ageing, atherosclerotic disease, and inflammation. ^6^ Moreover, periodontitis has also been associated with endothelial dysfunction. ^15^ ^,^ ^33^

Ligature-induced periodontitis in rats is among the most widely used experimental models of periodontitis. Alveolar bone loss, which is the main characteristic of periodontitis, is well-established seven days after ligature placement; ^2^ however, previous work from our group has demonstrated that systemic and vascular inflammation is more consistent from 14 days on. ^2^ ^,^ ^24^ Therefore, we performed our analysis two and three weeks after ligature placement. Two weeks after ligature placement, progressive bone loss was observed, suggesting that local inflammation was not resolved even after three weeks. This is corroborated by the increased levels of MPO two and three weeks after ligature placement. Two weeks after ligature placement, there was also endothelial dysfunction, agreeing with our previous results ^2^ and with human studies. ^15^ ^,^ ^33^ However, the reduced endothelium-dependent relaxation, which has been used as an indicator of endothelium function, was transient, and normal relaxation was present three weeks after ligature placement. ^24^ Therefore we have confirmed previous results showing that impairment on acetylcholine response induced by ligature in rats is a transient event. ^2^ ^,^ ^24^ Intriguingly, as shown by the increased levels of MPO, CRP and also by previous studies ^2^ ^,^ ^24^ , local and systemic inflammation is clearly ongoing by the time endothelium relaxation is restored. Thus, even with ongoing local and systemic inflammation, acetylcholine-induced relaxation is restored.

Endothelial function is generally evaluated by measuring arterial blood flow and vasodilation. Here, we evaluated change in blood pressure. However, we have previously demonstrated that the decrease in mean arterial pressure induced by acetylcholine and sodium nitroprusside is a valid approach to evaluate endothelial function. ^2^ ^,^ ^22^ The reduction in blood pressure induced by acetylcholine or sodium nitroprusside is primarily caused by transient dose-dependent vasodilation, without affecting the heart rate (data not shown). Thus, this decrease in mean arterial pressure results from a decrease in vascular resistance, and is not secondary to a cardiac effect. Moreover, arterial blood pressure is primarily determined by small resistance arteries, which are relevant in the initiation and development of cardiovascular diseases. ^13^

Endothelium-dependent relaxation in response to agonists or shear stress exerted by the flowing blood is generally attributed to the release of nitric oxide. ^35^ However, in some cases, particularly in small resistance type vessels, other mediators besides NO, such as EDH and/or PGI_2_, contribute to endothelium-dependent vasodilator response to agonists. ^7^ In endothelial cells, PGI_2_ is the main COX product. Prostacyclin is a potent vasodilator and its effect is mainly mediated through specific cell surface receptors, known as IP. ^18^ EDH is activated by an increase in intracellular calcium concentration in endothelial cells, followed by the opening of K_Ca_2.3 and K_Ca_3.1 channels, and the subsequent hyperpolarization of these cells ( Figure 1 ). The resulting endothelial hyperpolarization spreads via myoendothelial gap junctions, which then results in the hyperpolarization and relaxation of the smooth muscle. ^7^ ^,^ ^10^

With this in mind, we explored the role of these endothelium relaxant pathways in restoring acetylcholine response. It has been demonstrated that, in diabetic rats, despite increased oxidant stress, endothelium-dependent relaxation is maintained due to increased NOS-3 expression, resulting in increased NO synthesis. ^16^ Additionally, an increase in NOS-2 (iNOS) expression in the aorta of rats with periodontitis has recently been demonstrated. ^3^ However, given that the non-selective NOS inhibitor failed to change the acetylcholine response in ligature animals, NO does not appear to be involved in restoration of the acetylcholine response. Similarly, our group ^24^ and others ^3^ have demonstrated an up-regulation of COX- 2 in the vascular wall of ligature animals. However, indomethacin, a non-selective COX-inhibitor, has not been shown to change endothelium dependent responses. As indomethacin is not a very specific drug, presenting with other mechanisms aside from the inhibition of the COX enzyme, ^20^ we confirmed the results using highly selective COX-1 (SC-560) and COX-2 (NS-398) inhibitors. These inhibitors, whether used alone or together, also did not change the endothelium-dependent responses. Taken together, these results suggest that NO and COX products are not involved in the restoration of acetylcholine response.

EDH has been proposed to promote vasodilator response action through the initial activation of K_Ca_2.3 and K_Ca_3.1, which are present in the endothelium ^10^ and are sensitive to inhibition by a combination of apamin and TRAM-34. ^25^ ^,^ ^26^ Interestingly, the compensated response to acetylcholine 21 days after ligature placement was inhibited by the blockade of endothelial potassium channels with the combination of apamin plus TRAM-34. This suggests that endothelial calcium-activated potassium channels have a predominant involvement in restoring acetylcholine response. This also agrees with several *in vitro* studies on NOS-3 knockout mice, which report a compensatory increase in the activity of the EDH pathway in different vascular beds. ^12^ ^,^ ^32^ Some studies have also shown that K_Ca_3.1 channel expression is increased during vascular inflammation ^11^ .

Interestingly, none of the inhibitors used in this study were able to reduce the acetylcholine response in sham animals. The blockage of one or two pathways was not able to decrease the acetylcholine-induced reduction in blood pressure; however, blocking EDH, NOS, and COX simultaneously did reduce it (data not shown). This agrees with a large amount of literature showing that endothelium-dependent relaxation is redundant. ^14^ However, our results indicated that in ligature animals, which have deficient NO signaling, this balance is easily disrupted.

The results presented above indicate two important points. First, they show the importance of EDH for restoring endothelial relaxation, which probably provides vascular protection during the reduction of NO bioavailability. Second, these results show a limitation of endothelial-dependent relaxation as a marker of endothelial dysfunction. Endothelial-dependent vasomotion has been the most widely used clinical end point for assessment of endothelial function. However, the compensation in relaxation response by endothelial calcium-activated potassium channels could mask the reduction in NO bioavailability, therefore resulting in an increased risk of cardiovascular disease. It is noteworthy that NO has other important functions besides vasodilation, including thromboregulation, cell adhesion, and cell proliferation, ^21^ and these properties are not shared by EDH.

We did not evaluate the expression of potassium channels. Therefore, the precise mechanisms by which ligature-induced periodontitis mobilizes the endothelial calcium-activated potassium channels remain not entirely defined.

Given that ligature-induced periodontitis does not reproduce all aspects of human disease, and that the current study used a small number of animals and a short time frame of investigation, the results must be cautiously interpreted and carefully extrapolated to a clinical context.

## Conclusion

We confirmed our previous results showing that the impairment in endothelium-dependent hypotension during experimental periodontitis in rats is a transient event. The results show that restoration is not a consequence of resolution of the systemic inflammation, but of the development of a compensatory endothelium-dependent hypotension within 21 days after ligature-induced periodontitis. Moreover, we have demonstrated that this compensatory hypotension is mediated by the activation of endothelial calcium-activated potassium channels.
